# Polylysogeny magnifies competitiveness of a bacterial pathogen *in vivo*

**DOI:** 10.1111/eva.12243

**Published:** 2015-02-27

**Authors:** Nicola Burns, Chloe E James, Ellie Harrison

**Affiliations:** 1 Department of Biology, University of YorkYork, UK; 2 Biomedical Science Research Center, University of SalfordSalford, UK

**Keywords:** apparent competition, liverpool epidemic strain, polylysogeny, temperate phage

## Abstract

The rise of next generation sequencing is revealing a hidden diversity of temperate phages within the microbial community. While a handful of these phages have been well characterized, for the vast majority, the role of phage carriage, and especially multiple phage carriage, is poorly understood. The Liverpool epidemic strain of *Pseudomonas aeruginosa* is an aggressive pathogen in cystic fibrosis lung infections that has recently been found to contain several unique prophages within its genome. Here, we experimentally investigate the role of two of these phages *in vivo*, using an insect model of infection. We find that while no benefit is conferred by phage carriage in single bacterial infections, phages confer a large fitness advantage during mixed infections by mediating bacteria–bacteria competition. Differences between the two phages appeared to be associated with the rate at which the competitor acquired the phage, and therefore resistance. However, the advantage was greatest in the polylysogen, carrying both phages. These findings suggest that the LES phages may play an important role in host invasions and more generally show that the carriage of multiple phages may itself be beneficial by hindering the spread of resistance in rival bacterial populations.

## Introduction

Temperate bacteriophages (phages) are widespread and important viruses of bacteria. Unlike purely lytic phages, temperate phages can be transmitted both horizontally through the lytic cycle, and vertically through lysogeny, whereby the phage genome is inserted into the bacterial chromosome. This dual lifecycle confers several potential benefits to the host bacteria. Firstly, many phages carry useful bacterial accessory traits which often, although not exclusively (Mann et al. 2003), encode gene products associated with pathogenicity, such as toxins and antigenic molecules (Brussow et al. 2004). Secondly, phages may act as an anticompetitor mechanism: phages integrated into the chromosome (prophages) can undergo spontaneous induction and enter the lytic cycle, producing lytic phages able to infect and kill susceptible bacteria in the local area. While this results in the death of the individual host cell, it can provide a large competitive advantage for the remaining phage-carrying population, which are resistant to infection (Brown et al. 2006). Thirdly, the integration of phages into host genomes may itself act as a driver of genome innovation through increased mutagenesis and the supply of novel DNA (Goerke and Wolz 2010). Temperate phages therefore have the potential to play a major role in the ecology and evolution of bacterial populations.

Genome sequencing has revealed the presence of prophages in a large proportion of bacterial genomes, many of which carry multiple phages (polylysogens) (Figueroa-Bossi et al. 2001; Schuch and Fischetti 2009; Winstanley et al. 2009; Wang et al. 2010). While a small number of prophages have been identified as carrying specific, useful bacterial traits, the role of majority of these prophages, and in particular the benefits of polylysogeny, remains unclear. The Liverpool epidemic strain (LES) of *Pseudomonas aeruginosa*, a major pathogen in lung infections of cystic fibrosis (CF) sufferers, has been shown to carry multiple novel prophages of unknown function. Originally isolated in Liverpool, LES has been found to be extremely transmissible, infecting non-CF sufferers (McCallum et al. 2002) and spreading throughout hospitals in the UK and worldwide (Fothergill et al. 2012). It is also associated with increased patient morbidity (McCallum et al. 2002). Recent work has shown that disruption of these phages, through signature-tagged mutagenesis, leads to a significant reduction bacterial fitness in a rat model of chronic lung infection (Winstanley et al. 2009). Infective phage particles from four prophages have been detected in the sputa of CF sufferers (Fothergill et al. 2010; James et al. 2014), suggesting that these phages are actively entering the lytic cycle during infection. These results imply a role for these phages in LES infections; however, what benefit they confer is poorly understood.

In this study, we investigate the ecological consequences of single and multiple phage carriage using two temperate phages isolated from the LES strain in experimental infections using an insect model system; larvae of the greater wax moth (*Galleria mellonella*)*. Galleria* larvae have been successfully used as an *in vivo* model of both bacterial (Inglis et al. 2009; Yasmin et al. 2010; Evans and Rozen 2012; Hall et al. 2012) and fungal (Scully and Bidochka 2005) infection as they provide a tractable model host environment. Using this system we find that, in single-genotype infections, these phages appear to have no impact on the growth or virulence of their bacterial host. However, in mixed infections with phage-free bacteria, phage carriage, and especially multiple phage carriage, is highly beneficial. These results suggest that polylysogeny can in itself be beneficial to the bacterial host by mediating bacteria–bacteria competition.

## Methods

### Strain construction and culture conditions

To allow us to make direct comparisons between isogenic strains, experiments were conducted using a phage-free lab strain of *P. aeruginosa,* PAO-1, as the bacterial host. Two marked PAO-1 strains were produced following the methods of Koch et al. (2001); a focal strain PAO-1-*gm,* which carries a gentamycin resistance marker and a ‘reference’ strain PAO-1-*sm,* which carries a streptomycin resistance marker and was used as a competitor strain during competitive fitness assays. Phages LES*φ*2 and LES*φ*4 have been previously isolated from the LES strain LES-B58 (James et al. 2012). Single and double lysogens of the PAO-1-*gm* strain were produced following the methods of James et al. (2012), resulting in 3 lysogenic genotypes; PAO-1-*gm*-LES*φ*2 (from now on referred to as P2), PAO-1-*gm*-LES*φ*4 (P4), and PAO-1-*gm*-LES*φ*2 LES*φ*4 (P2&4). The nonlysogenic PAO-*gm* strain was used as the phage-free control strain (PF).

Wax moth larvae were obtained from wigglywigglers.com. Larvae were selected at random for inoculation and discarded if they showed any sign of melanization, which may indicate disease or the onset of pupation. Larvae were chilled briefly on ice prior to injection to reduce movement and the hind section dipped in ethanol to minimize contamination. To initiate infections, overnight bacterial cultures were diluted 100× in phosphate-buffered saline (PBS) and 5 μL (approx. 10^6^ bacteria) injected into the larvae using a disposable microsyringe. Larvae were incubated individually in separate sterile Petri dishes at 37°C.

### Growth rate and time-to-death assays

Four replicate overnight cultures were established for each of the four treatments, PF, P2, P4, and P2&4 making a total of 16 cultures. Each culture was used to inoculate seven larvae, in addition to which four larvae were injected with PBS only. Replicate larvae were then randomly split into seven groups with one group to be homogenized after 2, 4, 6, 8, 10, 12, and 24 h, respectively. At each time point, the homogenate was serially diluted and plated onto lysogeny broth (LB) agar containing gentamicin to determine bacterial density. In addition, the homogenate was filter sterilized to isolate free-phage, serially diluted in PBS and plated onto PAO-1 lawns. Throughout the experiment, the 24-h group was monitored for survival at hourly intervals. Larvae were scored as dead when there was no observable response when prodded lightly.

### Competitive fitness

Six replicate competitions were established for each treatment from a 1:1 mix of PA01-*sm* and either the PF, P2, P4, or P2&4 strain depending on the treatment. Therefore, 24 competitive cultures were established in total. These cultures were serially diluted and plated onto LB agar selecting for either streptomycin or gentamicin resistance to determine the bacterial density of the PA01-*sm* and treatment specific test strain, respectively, at 0 h.

To sample fitness at multiple time points each of the 24 cultures was diluted 100-fold in PBS, and three larvae were inoculated per replicate. After 6, 12, and 24 h, one of the three larvae, chosen at random, was homogenized, serially diluted, and the bacteria plated on to streptomycin- and gentamicin-selective media. Competitive fitness was estimated for each time point from the Malthusian parameters (Lenski et al. 1991).

In addition, we quantified the proportion of lysogens in the originally phage-free PA01-*sm* population by PCR screening. Ten streptomycin resistant clones from each competition were restreaked to remove contaminating free-phage particles, and colonies were screened using primers targeting LES*φ*2 (LES1nestF: TTTGGTGATGATCGGCTTAGC, LES1nestR: TGTGGAAGCGATCAGTCT) and LES*φ*4 (4tot1F: GCTCATGAGTGGCTGACAAC, 4tot1R: TCTTGGGCAGAGAACCATTC) (James et al. 2012).

### Statistical analysis

All analyses were conducted in R statistical package (R Foundation for Statistical Computing, Vienna, Austria). All data sets were analyzed in a fully factorial linear model and further investigated using Tukey's *post hoc* comparisons.

## Results

### Effects of lysogeny in single infections

**Figure 1:**
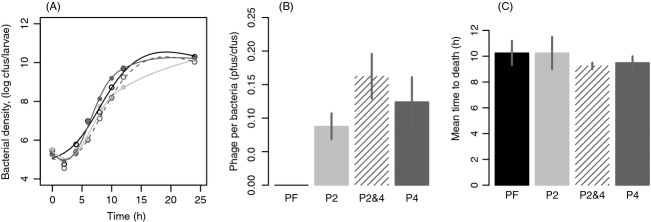
Life history of single infections of lysogenic strains. (A) Bacterial density thorough time during infection. Points show average bacterial density (*n* = 4) for the phage-free strain (black, empty), P2 (light gray, filled), P4 (dark gray, filled), P2&4 (dark gray, empty). Lines show splines predicted by a general additive model. (B) The average number of phage particles per bacteria during infection. As phage:bacteria ratio did not vary over time, bars show means of replicates (*n* = 4) averaged through time. Lines show standard error. (C) Average virulence of the four strains shown as mean time-to-death of larval hosts observed over a 24-h period (*n* = 4). Lines show standard error. Controls, inoculated with PBS only, are not shown as no deaths were observed for the duration of the experiment.

The impact of prophage carriage was first examined in single infections of each of the four strains. Average doubling time of the *in vivo* infections was 38.2 min (±1.3 SE), with no difference between the strains (Fig.1A: *F*_3,12_ = 1.02, *P* = 0.42). In all competitions-containing lysogens, infectious free-phage particles were present at an average frequency of 0.12 phage per bacteria (±0.02 SE), with no difference between strains (*F*_2,92_ = 1.01, *P* = 0.34), or through time (*F*_1,92_ = 1.02, *P* = 0.89).

The virulence of each strain in the wax moth larvae host was estimated by time-to-death assays by monitoring infected larvae hourly for 16 h. Control larvae that were inoculated with PBS remained alive after 24 h with no signs of illness. All larvae inoculated with bacteria became highly melanized and died within between 9 and 14 h, with no significant difference between strains (Fig.1C: *F*_3,12_ = 0.383, *P* = 0.77).

### Effects of lysogeny in mixed infection

**Figure 2:**
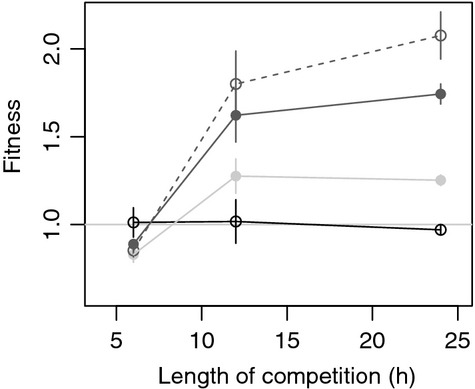
Relative fitness of lysogens in competition with an isogenic phage-free strain. Points show mean competitive fitness estimated after 6, 12, and 24 h of competition for the four different strains with a nonlysogenic competitor. A fitness of 1 indicates no difference in fitness from the competitor strain. Points show mean fitness of 6 replicates for treatments PF (black, empty), P2 (light gray, filled), P4 (dark gray, filled), and P2&4 (dark gray, empty). Error bars denote standard error.

The relative fitness of the four strains was examined in competition with a labeled, phage-free isogenic competitor. As the competitor strain is susceptible to infection and can therefore become lysogenized during the competition, fitness was estimated after 6, 12, and 24 h to examine the effect of time on this relationship. Competitive fitness of the nonlysogenic PF strain relative to the test strain (which is isogenic with the exception of the antibiotic resistance markers) maintained a fitness not significantly different from 1 (Fig.2: *F*_1,16_ = −0.15, *P* = 0.7) with no variation through time (*t*_11_ = −0.41, *P* = 0.69) demonstrating that neither marker alters fitness relative to the other.

In contrast, the three lysogenic strains showed different fitness profiles both through time and relative to each other (STRAIN X TIME: *F*_2,48_ = 4.14, *P* = 0.022). At 6 h, fitness estimates for each of the lysogenic strains was <1 (on average 0.85 ± 2.06%), indicating a fitness cost relative to the plasmid-free strain. No significant difference between the three lysogenic strains was observed at this time point (for each pairwise *post hoc* comparison *P* > 0.851), but both the fitness of the P2 and the fitness of the P2&4 strains were significantly reduced relative to the nonlysogenic PF strain (*t*_20_ = −2.48, *P* = 0.022 and *t*_20_ = −2.16, *P* = 0.043, respectively). After 12 h, however, this fitness interaction was reversed and all three strains had an average fitness >1. By 24 h, the three lysogenic strains all exhibited a large significant fitness advantage relative to the test strain (P2: *t* = 2.645, *P* = 0.0155; P4: *t* = 7.243, *P* < 0.0001; and P2&4: *t* = 10.363, *P* < 0.0001). This fitness advantage varied widely between strains. Among the two single lysogens, P4 had a significantly larger fitness advantage than P2 (1.74 ± 0.057% compared to 1.25 ± 0.030%, *t* = 4.599, *P* < 0.001). However, the double lysogen, P2&4, had a fitness advantage of 2.07 ± 0.135% indicating a 100% fitness advantage over the phage-free strain, and significantly larger than both single lysogens (compared to P2: *t* = 7.719, *P* < 0.001 and P4: *t* = −3.120, *P* = 0.025).

### Lysogenic conversion during competition

**Figure 3:**
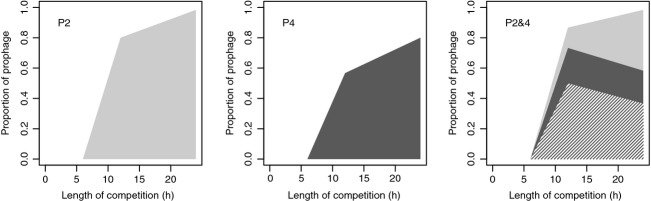
Rate of lysogeny during competition. The average proportion of lysogens in the initially nonlysogneic PAO-1-*sm* population during competition with lysogens P2, P4, and P2&4 (left to right). Colors denote the proportion of lysogens carrying LES*φ*2 (light gray), LES*φ*4 (dark gray), and both LES*φ*2 and LES*φ*4 prophages (shaded). Values shown are the means of six populations. For each population, the proportion of lysogens was estimated by screening 10 clones for the presence or absence of phages.

The proportion of the lysogens among the initially susceptible competitor population was estimated by PCR for 10 clones per competition at each time point (Fig.3). After 6 h, no lysogens were identified from any of the competitions. After 12 h, however, lysogens were identified in all populations of each treatment. When comparing the total proportion of lysogens (i.e., the proportion of clones carrying at least one prophage), both the main effects of time and strain were significant (TIME *F*_2,50_ = 4.22, *P* = 0.0202, and STRAIN *F*_1,50_ = 86.71, *P* < 0.0001). After 24 h, between 90% and 100% of competitor clones from both the P2 and the P2&4 treatments carried at least one prophage. In comparison, prophages were identified in an average of 80.0% (±8.6% SE) of clones from competitions with the P4 strain (*t*_6.11_ = −2.98, *P* = 0.024). In the double lysogen, however, not all lysogenic competitor clones carried both prophages. Between the six replicate competitions run for 24 h between 20% (2/10) and 50% (5/10) of clones screened carried both phages.

## Discussion

Many bacterial strains carry multiple ‘cryptic’ prophages whose function remains largely unknown. Using an insect model of infection, we have investigated the role of two such phages, LES*φ*2 and LES*φ*4, in the success of the aggressive LES strain of *P. aeruginosa in vivo*. We find that both phages confer a large fitness benefit to their bacterial hosts during competition with susceptible bacteria. However, this benefit does not appear to be due to any intrinsic advantage of phage carriage; no differences in growth rates or virulence were identified between lysogens and their phage-free counterpart under the conditions of this study. While we are unable to exclude the possibility that, within the native lung environment, these phages may provide additional benefits not expressed in the wax moth larvae, these results clearly demonstrate a large competitive advantage due to ‘apparent competition’, mediated by phages in their infectious state, which are able to infect and lyse susceptible nonlysogens. This is also supported by the finding that in the initial stages of infection (i.e., after 6 h of competition), lysogens were not beneficial and in fact were associated with a ∼10% fitness cost relative to the nonlysogen. This can be explained by the fact that the benefits of producing phages in their lytic form (i.e., lysis of competitors) will be density dependent and therefore greater in the latter stages of infection where the encounter rate between infectious phage particles and susceptible bacteria is high. The cost of phage carriage, which may include the biosynthetic burden of carrying additional DNA (Glick 1995; Rozkov et al. 2004) and cell death through spontaneous induction, is paid regardless of the presence of susceptible hosts, therefore potentially explaining the cost observed over shorter time scales.

Our results also revealed a marked difference in the scale of the fitness advantage conferred by the different phages and between single and multiple phage infections. Lysogens carrying LES*φ*2 had significantly lower fitness than those carrying the LES*φ*4 phage; however, no difference was observed in the growth rate, virulence, or the proportion of infectious phage particles released during growth. This difference instead appears to be associated with the ability of these phages to lysogenize the competitor strain, which has previously been shown to be a major determinant of the benefit of phage carriage (Gama et al. 2013). Among single lysogens, the proportion of lysogenized competitor clones was higher in the P2 competitions compared to those in competition with P4 lysogens. Rapid lysogenization of the competitor effectively ‘levels the playing field’ between the strains earlier in the competition, reducing the benefit of phage carriage to original host strain.

The largest fitness benefit (>100%) was observed in the double lysogen. During competition with this strain, nearly 100% of the competitor strain became lysogenized; however, on average, <50% carried both prophages and were therefore completely resistant to lysis. Previous work has shown that single lysogens of the LES*φ*4 phage are fully susceptible to infection by LES*φ*2, while LES*φ*2 carrying strains are partially, although not completely resistant to LES*φ*4 (James et al. 2012), therefore making a large proportion of the population susceptible to lysis. These results suggest that polylysogeny, which is observed commonly among pathogenic bacteria (Figueroa-Bossi et al. 2001; Schuch and Fischetti 2009; Winstanley et al. 2009; Wang et al. 2010), may in itself be adaptive for the bacterial host as it hinders the process of immunization of competitor strains and prolongs the effectiveness of a lysogen's ‘armory’ of viruses.

This relationship between polylysogeny and host fitness also has interesting consequences for the phages themselves. As integrated elements prophages gain a benefit from polylysogeny through increased fitness of their host; therefore, it may be imagined that between-host selection may favor phages that accommodate superinfection. However, polylysogeny may also have fitness costs for the phages themselves. Most significantly, prophages which cohabit hosts containing more virulent phages (i.e., those that are more likely to initiate lysis and/or are able to replicate faster) are at a significant numeric disadvantage (Refardt 2011). Therefore, selection occurring within-hosts (i.e., between phages) may in fact favor more aggressive phages or resistance to superinfection. The LES phages themselves display a hierarchy (James et al. 2012) of resistance which may be indicative of this conflict.

These findings therefore suggest that the LES phages are effectively acting as weapons in bacterial warfare. Recent work has shown that lytic LES particles are consistently released throughout long-term lung infections, implying that these phages are playing a significant role in ecology of this chronic infection (James et al. 2014). Similar results have been identified in several other bacterial pathogens, namely *Escherichia coli* (Brown et al. 2006; Gama et al. 2013), *Bordetella* (Joo et al. 2006) and *Salmonella* (Bossi et al. 2003) and *Enterococcus faecalis* (Duerkop et al. 2012) suggesting that phage-mediated competition may be a common strategy employed by pathogenic bacteria. More broadly, temperate phages form part of a wider group of horizontally transmitted elements that influence host fitness through apparent competition including plasmids and integrative elements which encode genes for bacteriocins (Riley and Wertz 2002). The benefits of carrying such elements, however, are likely to be short-lived as susceptible bacteria can become infected, negating the competitive advantage to the original hosts, suggesting that these elements are particularly important in the initial stages of invasion of new environments already occupied by resident bacteria or in repelling potential invading bacteria. The carriage of multiple phages may be one strategy employed to get around this short-coming, as multiple steps are required for immunization to occur, and thus, the benefit of the phage ‘armory’ is prolonged. This finding suggests that ‘cryptic’ phages may in fact be playing a major role in the ecology and evolution of bacterial pathogens.

## Data archiving statement

Data available from the Dryad Digital Repository: http://doi.org/10.5061/dryad.5b58js.
